# Detection of metallo-β-lactamases-encoding genes among clinical isolates of *Pseudomonas aeruginosa* in a tertiary care hospital, Kathmandu, Nepal

**DOI:** 10.1186/s13104-017-3068-9

**Published:** 2017-12-08

**Authors:** Mahesh Acharya, Prabhu R. Joshi, Kamal Thapa, Rajan Aryal, Trishna Kakshapati, Supriya Sharma

**Affiliations:** 1 Central Department of Microbiology, Tribhuvan University, Kirtipur, Kathmandu, Nepal; 2 Kathmandu College of Science and Technology, Tribhuvan University, Kathmandu, Nepal; 3 Kantipur College of Medical Sciences, Tribhuvan University, Kathmandu, Nepal; 4 Annapurna Neurological Institute and Allied Sciences, Kathmandu, Nepal

**Keywords:** Metallo-β-lactamases, Imipenem-nonsusceptible, *bla*_VIM-2_, *bla*_IMP-1_

## Abstract

**Objectives:**

This study was carried out to determine the prevalence of metallo-β-lactamases (MBLs) producing *Pseudomonas aeruginosa* in imipenem-nonsusceptible isolates and to detect MBL-encoding genes among MBLs-positive isolates.

**Results:**

Metallo-β-lactamases production was detected in 68.6% isolates of *P*. *aeruginosa* with reduced susceptibility to imipenem. The *bla*_VIM-2_ gene was detected in 75% isolates and *bla*_IMP-1_ was detected in 25% isolates. All MBLs-positive isolates were multidrug resistant with a high level of resistance to imipenem (MIC 16 to ≥ 32 µg/ml), meropenem (MIC 16 to ≥ 32 µg/ml), and ceftazidime (MIC 64 to ≥ 512 µg/ml). All MBL-positive isolates were susceptible (MIC ≤ 2 µg/ml) to colistin. We found high prevalence of MBL-producing *P*. *aeruginosa*. To our knowledge this is the first report of detection of *bla*_VIM-2_ and *bla*_IMP-1_ in *P. aeruginosa* from Nepal. This indicates the need for awareness to prevent the spreading of these resistant isolates in hospital setting.

## Introduction

*Pseudomonas aeruginosa* is a Gram-negative, non-fermentative organism found in diverse environmental settings [1]. It is an opportunistic pathogen, causing serious infection in patients with weakened immune systems [2]. This organism is generally intrinsically resistant to a wide variety of antimicrobial agents as well as it has the capacity to develop resistance by mutation or acquisition of foreign resistance genes against different antibiotic classes [3]. Carbapenem, including imipenem, meropenem and doripenem are often used as a last resort for treatment of infections caused by *P. aeruginosa* and other Gram-negative bacteria [4, 5]. However, carbapenem-resistant *P. aeruginosa* has become prevalent globally [6, 7]. Carbapenem resistance may arise in *P. aeruginosa* via OprD channel deficiencies, up-regulation of efflux pumps and production of various kinds of carbapenemases, including serine β-lactamases of Ambler classes A and D and metallo-β-lactamases (MBLs) of Ambler class B [8].

Among various mechanism of resistance for carbapenem in *P. aeruginosa*, production of MBLs is of particular concern because of their rapid spread, potent carbapenemase activity, resistance to β-lactamase inhibitors and ability to hydrolyze all β-lactam antibiotics with the exception of aztreonam [9]. Furthermore, MBLs encoding genes are usually located on integrons, the mobile genetic elements that also carry genes encoding for resistance to aminoglycoside and other antibiotics resulting in multidrug resistance (MDR) [10].

Several types of MBLs, such as IMP, VIM, SPM, GIM, AIM, FIM and NDM and their variants have been identified in *P. aeruginosa* [11–13]. Among them, variants of VIM and IMP types such as *bla*_VIM-1_, *bla*_VIM-2_ *, bla*_IMP-1_ and *bla*_IMP-2_ are the most commonly found MBL genes in *P. aeruginosa* and responsible for many nosocomial outbreaks [9].

Although, production of MBLs in clinical isolates is a serious therapeutic problem, so far limited information is available on MBL production in *P. aeruginosa* clinical isolates from Nepal. The objectives of this study were to determine the prevalence of MBL producing *P*. *aeruginosa* and to detect MBL-encoding genes (*bla*_VIM-1_, *bla*_VIM-2_ *, bla*_IMP-1_ and *bla*_IMP-2_).

## Main text

### Methods

#### Bacterial isolates

Clinical specimens submitted for routine culture and antibiotic susceptibility testing from hospitalized patients during the period of October 2015 to July 2016, at the microbiology laboratory of the Annapurna Neurological Institute and Allied Sciences were processed. *P*. *aeruginosa* were isolated and identified based on conventional biochemical test [14]. All *P*. *aeruginosa* isolates were screened for reduced susceptibility to imipenem (MIC ≥ 8 µg/ml) by E-test strips.

#### Confirmation of MBL production and antimicrobial susceptibility testing

All the isolates that showed reduced susceptibility to imipenem were tested for MBL production by the imipenem-EDTA disk diffusion test [15]. Susceptibility testing of the isolates were performed by disk diffusion according to the Guidelines of Clinical and Laboratory Standard Institute (CLSI) [16]. The following antimicrobial agents were used: ceftazidime (30 µg), imipenem (10 µg), meropenem (10 µg), ciprofloxacin (5 µg), aztreonam (30 µg), amikacin (30 µg), gentamicin (10 µg), piperacillin (100 µg), piperacillin–tazobactam (100/10 µg) and cefepime (30 µg). MIC of imipenem, meropenem, ceftazidime and colistin were determined by E-test strips (according to manufacturers instruction) and agar dilution method on all MBLs-producing isolates [16]. All the antibiotics and E-test strips were procured from Hi-media, India. *P. aeruginosa* ATCC 27853 was used as a quality control in the susceptibility testing.

#### Detection of MBL encoding genes by multiplex PCR

Total DNAs of MBL-positive *P. aeruginosa* isolates were extracted using a boiling method, briefly; 3–5 colonies from an overnight culture grown on Mueller–Hinton agar (Hi-media, India) were transferred to 300 µl of nuclease-free water and boiled at 100 °C for 10 min. After centrifugation at 12,000×*g* for 5 min, the supernatant was used as a source of template for amplification. Multiplex PCR amplification of MBL genes including *bla*_VIM-1_, *bla*_VIM-2_, *bla*_IMP-1_ and *bla*_IMP-2_ were performed (5 Prime/02, Bibby Scientific, UK) [17]. Amplification was carried out with the following thermal cycling conditions: initial denaturation at 95 °C for 5 min and 36 cycles of amplification consisting of 40 s at 94 °C, 40 s at 52 °C, 50 s at 72 °C, with 5 min at 72 °C for the final extension.

*Pseudomonas aeruginosa* NCTC 13437 producing *bla*_VIM_ and *Escherichia coli* NCTC 13476 producing *bla*_IMP_ kindly provided by Prof. Neil Woodford from Public Health England were used as positive control and *P*. *aeruginosa* ATCC 27853 were used as negative control.

### Results

A total of 98 *P. aeruginosa* were obtained during the study period. Of the total isolates, only 35 (35.7%) isolates showed reduced susceptibility to imipenem (MIC ≥ 8 µg/ml). Of the 35 isolates with reduced susceptibility to imipenem, 24 (68.6%) were positive for MBL production by the imipenem-EDTA disk diffusion test. Eighteen isolates (75%) were *bla*_VIM-2_ positive and 6 (25%) were *bla*_IMP-1_ positive. In this study, *bla*_VIM-1_ and *bla*_IMP-2_ MBL allele were not detected. MBL-positive isolates were from intensive care unit (ICU) 14 (58.4%) and 10 (41.6%) were from general ward patients.

All MBL positive isolates were susceptible (MIC ≤ 2 µg/ml) to colistin and majority of MBL-producing isolates were also susceptible to aztreonam (62.5%), whereas 24 (100%) were resistant to meropenem, piperacillin, ceftazidime and cefepime. All MBL-positive isolates had high resistance rates to ciprofloxacin (75%), amikacin (83.4%) gentamicin (91.7%) and piperacillin-tazobactam (95.8%). Using Magiorakos et al. definitions for multidrug-resistant and panresistant *P. aeruginosa* [18], all MBL positive isolates were multidrug resistant with a high level of resistant to imipenem (MIC 16 to ≥ 32 µg/ml), meropenem (MIC 16 to ≥ 32 µg/ml), and ceftazidime (MIC 64 to ≥ 512 µg/ml). Characteristics of MBL-producing *P*. *aeruginosa* are shown in (Table 1).

**Table 1:** Characteristics of metallo-β-lactamases producing *P*. *aeruginosa*

Isolate	Hospital site	Sample	*bla*_VIM-2_	*bla*_IMP-1_	MICs (µg/ml)			
					IPM	MRP	CAZ	CL
1	ICU	Sputum	+	−	16	32	256	1
2	ICU	Sputum	+	−	64	64	512	1
3	GW	Urine	+	−	128	128	512	0.5
4	ICU	Sputum	−	+	128	128	256	2
5	GW	Urine	+	−	128	256	512	2
6	GW	Urine	+	−	16	16	512	0.5
7	GW	Sputum	−	+	256	128	512	2
8	ICU	Pus/wound swab	−	+	64	64	512	1
9	GW	Urine	−	+	128	64	64	2
10	GW	Sputum	−	+	128	64	128	0.25
11	ICU	Tracheal aspirates	+	−	256	64	128	1
12	ICU	Urine	+	−	256	64	256	1
13	ICU	Sputum	+	−	128	64	256	1
14	ICU	Sputum	+	−	32	32	512	0.25
15	ICU	Pus/wound swab	+	−	64	32	128	2
16	GW	Urine	+	−	128	128	256	2
17	ICU	Sputum	+	−	128	128	512	0.5
18	ICU	Sputum	+	−	128	128	256	2
19	GW	Pus/wound swab	+	−	64	64	512	2
20	GW	Urine	+	−	256	128	128	2
21	GW	Sputum	+	−	16	32	256	1
22	ICU	Urine	−	+	128	32	64	1
23	ICU	Tracheal aspirates	+	−	256	256	512	2
24	ICU	Sputum	+	−	128	256	256	1

*ICU* intensive care unit, *GW* general ward, *MIC* minimum inhibitory concentration, *IPM* imipenem, *MRP* meropenem, *CAZ* ceftazidime, *CL* colistin

### Discussion

Our study showed that 68.6% (24/35) isolates of *P*. *aeruginosa* with reduced susceptibility to imipenem were MBL producers. This finding was higher than those reported in the previous study from Nepal [19], indicating that MBL-producing *P*. *aeruginosa* is increasing. High prevalence of MBL producing *P. aeruginosa* was also detected in India, a neighboring country of Nepal (69.8%) [20], Taiwan (55.1%) [21], and Egypt (68.7%) [22]. The reduced imipenem susceptibilities in MBL-negative isolates might be due to other mechanisms such as deficiency in porin channel combined with production of AmpC β-lactamases [8].

In the present study, *bla*_VIM-2_ was the most commonly detectable genes among the different MBL genes analyzed; 75% of MBL-positive isolates carried *bla*_VIM-2_. Only 25% of MBL-positive isolates carried *bla*_IMP-1_. This finding was supported by result of previous studies from around the world demonstrating *bla*_VIM-2_ as the most common MBL genes responsible for reduced susceptibility to imipenem in *P*. *aeruginosa* and pose greatest clinical threat [23]. *bla*_VIM-2_ gene is associated with many nosocomial outbreaks due to MBL-producing *P*. *aeruginosa* [24]. Although, less than VIM-2 type, IMP-1 also have been reported from worldwide [25]. Due to the high mortality rate of infections caused by MBL-producing *P*. *aeruginosa*, their presence in hospital setting is of great concern. To evaluate the threat of MBL-producing *P. aeruginosa* and its associated risk factors, well-designed multicentre epidemiological studies are urgently required.

In this study, all MBL-producing isolates were MDR, leaving the limited treatment option. Based on the in vitro testing; the most effective antibiotic against MBL-producing isolates were colistin (100% susceptible, MIC ≤ 2 µg/ml), followed by aztreonam (62.5% susceptible). The existence of the MBL genes on integrons together with other resistance gene makes the MBL-positive isolates MDR [10]. Due to the limited treatment option (potentially toxic polymyxin B and colistin) for the infections caused by MBL-producing isolates and the mobile nature of MBL, early detection of MBL-producing isolates in hospital setting is necessary to initiate the appropriate infection control procedure to avoid the spread of these multidrug resistant isolates.

Aztreonam is stable against MBLs [10]; however in this study 37.5% of the MBL-producing isolates were resistant to aztreonam, suggesting a possible association with other resistance mechanisms such as AmpC type β-lactamases or ESBLs [1].

Many studies have shown that intensive care unit is the epicenter and the main source of amplification and dissemination of antimicrobial resistance [26], a finding that was also observed in this study among the 24 MBL-positive isolates; 14 (58.4%) were isolated from ICU while 10 (41.6%) were from general ward patients. Since more than 50% of MBL-producing *P*. *aeruginosa* isolates have been recovered from ICU, it is highly probable that it might be a clonal spread. Unfortunately, because of some limitations, such as lack of more equipped laboratory, we were not able to do genotypic comparisons of the isolates.

### Conclusion

In conclusion, our study showed the presence of *bla*_VIM-2_ and *bla*_IMP-1_ producing *P. aeruginosa* for the first time in Nepal that underscores the necessity of screening all imipenem-resistant isolates for MBL production and implementation of infection control programs to prevent spread of such organisms.

### Limitations

- The study was unable to determine whether these MBL genes are transferable or not.
- The study was unable to investigate other MBL-encoding genes such as NDM-1.
- Due to the lack of laboratory equipment, we could not perform genotypic comparisons of the isolates.

## Abbreviations

ATCC: American Type Culture Collection
CAZ: ceftazidime
CL: colistin
EDTA: ethylenediaminetetraacetic acid
GW: general ward
ICU: intensive care unit
IPM: imipenem
MBL: metallo-β-lactamase
MDR: multidrug resistance
MIC: minimum inhibitory concentration
MRP: meropenem
NCTC: National Collection of Type Cultures
OPD: out patients department
